# By reducing hexokinase 2, resveratrol induces apoptosis in HCC cells addicted to aerobic glycolysis and inhibits tumor growth in mice

**DOI:** 10.18632/oncotarget.3800

**Published:** 2015-04-12

**Authors:** Weiqi Dai, Fan Wang, Jie Lu, Yujing Xia, Lei He, Kan Chen, Jingjing Li, Sainan Li, Tong Liu, Yuanyuan Zheng, Jianrong Wang, Wenxia Lu, Yuqing Zhou, Qin Yin, Huerxidan Abudumijiti, Rongxia Chen, Rong Zhang, Li Zhou, Zheng Zhou, Rong Zhu, Jing Yang, Chengfen Wang, Huawei Zhang, Yingqun Zhou, Ling Xu, Chuanyong Guo

**Affiliations:** ^1^ Department of Gastroenterology, Shanghai Tenth People's Hospital, Tongji University School of Medicine, Shanghai, China; ^2^ The First Clinical Medical College of Nanjing Medical University, Nanjing, China; ^3^ The First Affiliated Hospital of Soochow University, Suzhou, China; ^4^ Department of Gastroenterology, Shanghai Tongren Hospital, Jiaotong University of Medicine, Shanghai, China

**Keywords:** resveratrol, hexokinase 2, hepatocellular carcinoma, apoptosis

## Abstract

Cancer cells exhibit an altered metabolic phenotype known as the aerobic glycolysis. The expression of HK2 changes the metabolic phenotype of cells to support cancerous growth. In the present study, we investigated the inhibitory effect of resveratrol on HK2 expression and hepatocellular carcinoma (HCC) cell glycolysis. Aerobic glycolysis was observed in four HCC cell lines compared to the normal hepatic cells. Resveratrol sensitized aerobic glycolytic HCC cells to apoptosis, and this effect was attenuated by glycolytic inhibitors. The induction of mitochondrial apoptosis was associated with the decrease of HK2 expression by resveratrol in HCC cells. In addition, resveratrol enhanced sorafenib induced cell growth inhibition in aerobic glycolytic HCC cells. Combination treatment with both reagents inhibited the growth and promoted apoptosis of HCC-bearing mice. The reduction of HK2 by resveratrol provides a new dimension to clinical HCC therapies aimed at preventing disease progression.

## INTRODUCTION

One of the metabolic features of tumor cells is the aerobic glycolysis, by which tumor cells, even in the presence of sufficient oxygen, activate glycolysis and convert pyruvate to lactate acid rather than totally oxidizing it by Krebs cycle [1]. Therefore, glycolysis, the major pathway for energy production, is pivotal for the survival and growth of cancer cells [2]. The mechanisms underlying the shift from the Krebs cycle to glycolysis in cancer cells are well studied, and it is believed that hexokinase 2 (HK2) contributes to this process [3].

The first important irreversible step of glycolysis is catalyzed by HKs, which phosphorylate glucose to glucose-6-phosphate. Four HK isozymes have been identified in mammals and are known as HK1, 2, 3, and 4 [4]. HK1 and HK2 bind to the mitochondrial voltage dependent anion channel (VDAC) through their hydrophobic N terminal. Disruption of the binding of HK with VDAC causes mitochondrial dysfunction and induces cell death [5]. In human carcinomas, including hepatocellular carcinoma (HCC), HK2 is highly expressed to produce more energy to support accelerated growth [6]. The upregulated mitochondria-bound HK2 in tumor cells, as well as the high glucose phosphorylation activity, promote tumor cell proliferation and survival by enhancing energy production and the escape from mitochondria-associated cell death [7]. Therefore, HK2 in particular, is regarded as a potential target for anticancer therapy, and the screening of drugs that suppress HK2 expression and prevent metabolic transformation becomes meaningful [5].

Resveratrol (3,4,5-trihydroxy-trans-stilbene) is a dietary polyphenol derived from grapes, berries, peanuts, and other sources [8]. Resveratrol has anticancer activity at multiple stages of tumor development and progression, and it has minimal toxicity to normal cells. The chemo-preventive effect of resveratrol has been shown in a variety of cancer cell lines, including HeLa, A549, and MCF-7 cells [9, 10]. Previous studies have proposed different mechanisms to explain the anti-proliferative effect of resveratrol, including the upregulation of tumor suppressor genes such as BRCA1/2 [11] and p53 [12], and the phosphorylation of Rb and transcription factors such as AP-1 and NF-kB [13]. Resveratrol was recently suggested to inhibit cellular survival through SIRT1-associated AMPK activation [14]. Resveratrol is also known to inhibit mTOR, which is one of the central metabolic integrators [15, 16]. In addition, resveratrol can suppress glycolysis by inhibiting PI3K signaling, blocking the cell cycle in B cell lymphomas [17]. However, the effect of resveratrol on the expression of HK2 during the metabolic transformation of HCC cells has not been analyzed to date. In the presented study, we show that the metabolic phenotype of HCC cells is characterized by glucose to lactate conversion and suppressed oxidative activity. Resveratrol inhibits glycolysis and induces apoptosis in HCC cells. Further investigation of the underlying mechanism showed that resveratrol inhibits HK2 expression and activates mitochondria-associated apoptosis. In addition, resveratrol improved the resistance of aerobic glycolytic HCC cells to the multikinase inhibitor sorafenib, the only clinically effective drug that can slightly prolong the survival of HCC patients. The results of the present study suggest that improving our understanding of the mechanisms underlying the effect of resveratrol on tumor proliferation and metabolism may help identify more effective treatments for patients with HCC.

## RESULTS

### Metabolic characterization of HCC cell lines

**Figure 1:**
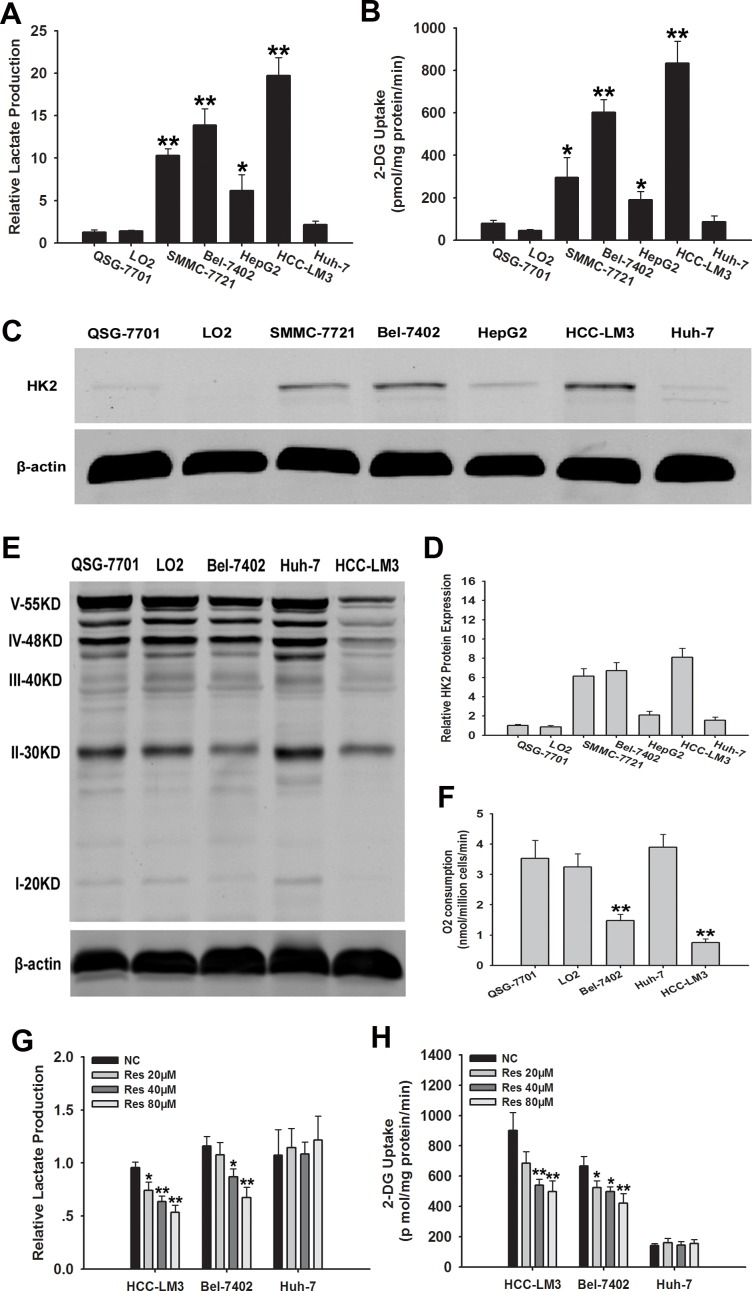
Metabolic features and effects of resveratrol on glycolysis in HCC cell lines (**A** and **B**) Normalized lactate production and 2-DG uptake in HCC cells (SMMC-7721, Bel-7402, HepG2, HCC-LM3, Huh-7), and normal hepatic cells (QSG-7701 and LO2) within 72 h of culture under normoxic conditions. The lactate production was normalized to the level of QSG-7701 cell line. (**C**-**E**) Western blot analysis of HK2 and OXPHOS in total cell extracts from HCC cells and normal liver cells. β-actin was used as a loading control. The histogram represents the results of HK2 staining in three independent experiments (means ± s.e.m.). (**F**) O_2_ consumption in the indicated cell lines (nmol O_2_/million cells/min) was tested by a Clark-type oxygen electrode, which detected the concentration of dissolved oxygen in a closed chamber over time. (**G** and **H**) Relative lactate production and 2-DG uptake from HCC cell lines (HCC-LM3, Bel-7402, and Huh-7) in the absence or presence of resveratrol (20, 40, and 80 μM) within 72 h of culture under normoxic conditions. 2-DG uptake, lactate production, and O_2_ consumption were performed in triplicate and data represent the mean ± s.e.m. (*P < 0.05; **P < 0.01).

The metabolic characteristics of five HCC cell lines were compared with those of two normal hepatic cell lines (QSG-7701 and LO2) cultured under normoxic conditions for 72 h. Glucose uptake and lactate production were compared between HCC cell lines showing heterogeneity in cellular metabolism (Fig. 1A and 1B). The concentration of lactate in the cell culture supernatant was significantly higher in HCC-LM3, SMMC-7721, and Bel-7402 cells than in QSG-7701 and LO2 cells. The concentration of lactate was slightly increased in HepG2 cells compared to QSG-7701 and LO2 cells (*P* < 0.05). In addition, the cellular glucose uptake was markedly potentiated in HCC-LM3 and Bel-7402 cells compared with QSG-7701 and LO2 cells, (834 vs. 602 pmol/mg/min of 2-DG, respectively). Glucose uptake was slightly higher in SMMC-7721 and HepG2 cells than in QSG-7701 and LO2 cells, whereas Huh-7 cells showed no glucose uptake changes in lactate production and glucose uptake. These results indicate that HCC cell lines show an increased rate of aerobic glycolysis compared to healthy cells.

Our data showed that glycolysis was used as a bioenergetic pathway in more than 80% of our tested HCC cell lines. The first rate-limiting step is the conversion of glucose to glucose 6-phosphate (G-6-P) during aerobic glycolysis catalyzed by HK, which is the key mediator of glucose metabolism. Therefore, HK2 expression was assessed by western blotting in two healthy liver cell lines and five HCC cell lines (Fig. 1C and 1D). At least four of HCC cell lines (HCC-LM3, Bel-7402, SMMC-7721, and HepG2) expressed HK2, whereas Huh-7 and normal liver cells did not. HK2 was expressed exclusively in the high-glycolytic HCC-LM3 and Bel-7402 cell lines, but not in the low-glycolytic Huh-7 cell line. Based on these results, HCC-LM3 and Bel-7402 cells, which showed the highest aerobic glycolysis rate of all the HCC cell lines tested, were selected as typical inherent aerobic glycolytic HCC cell lines with high HK2 expression, and Huh-7 was selected as a representative low-glycolytic HCC cell line, showing low glucose to lactate conversion. These cell lines were used for subsequent experiments.

In tumor cells, the aerobic glycolysis is generally correlated to decreased oxygen consumption, which results from disrupted oxidative phosphorylation (OXPHOS) capacity in mitochondria [25]. We therefore tested byproducts of OXPHOS metabolism and O_2_ consumption to determine whether representative high- and low-glycolytic HCC cell lines showed differences in OXPHOS capacity and oxygen consumption (Fig. 1E and 1F). Consistent with our hypothesis, OXPHOS metabolism-correlated proteins, denoted as complexes I/II/III/IV/V in the electron transport chain, were markedly decreased in the representative aerobic glycolytic HCC cell lines (HCC-LM3 and Bel-7402), showing approximately 2.5-fold lower levels than in the low-glycolytic HCC cell line (Huh-7) and healthy cells (QSG-7701 and LO2). Moreover, O_2_ consumption, which reflects the level of OXPHOS metabolism, was lower in representative aerobic glycolytic HCC cells (HCC-LM3 and Bel-7402) than in the other cells examined (Fig. 1F).

### Resveratrol inhibits glycolysis in aerobic glycolytic HCC cell lines

Because resveratrol suppresses aerobic glycolysis in several cancers, including breast and ovarian cancers [26, 27], we examined the ability of resveratrol to induce similar changes in HCC cell lines. Our data showed that resveratrol (20 μM) treatment of HCC-LM3 cells significantly decreased the concentration of lactate in the cell culture supernatant (*P* = 0.018) compared to that in the untreated control. Bel-7402 cells treated with resveratrol (40 μM) showed significantly lower lactate concentration (*P* = 0.012) than the untreated control group. Moreover, increasing doses of resveratrol decreased lactate concentration in the cell culture media in both the HCC-LM3 and Bel-7402 cell lines (Fig. 1G).

HCC-LM3 cells treated with resveratrol (40 μM) showed significantly lower (*P* = 0.008) glucose uptake (541 pmol/mg/min) than the untreated control (901 pmol/mg/min). In Bel-7402 cells, resveratrol (20 μM) also led to markedly lower (*P* = 0.031) glucose uptake (524 pmol/mg/min) compared to the untreated control (668 pmol/mg/min; Fig. 1H). By contrast, resveratrol treatment did not change the lactate level and glucose uptake of the low-glycolytic Huh-7 cell line (Fig. 1G and 1H).

### Resveratrol inhibits proliferation and induces apoptosis partly by suppressing HCC glycolysis

**Figure 2:**
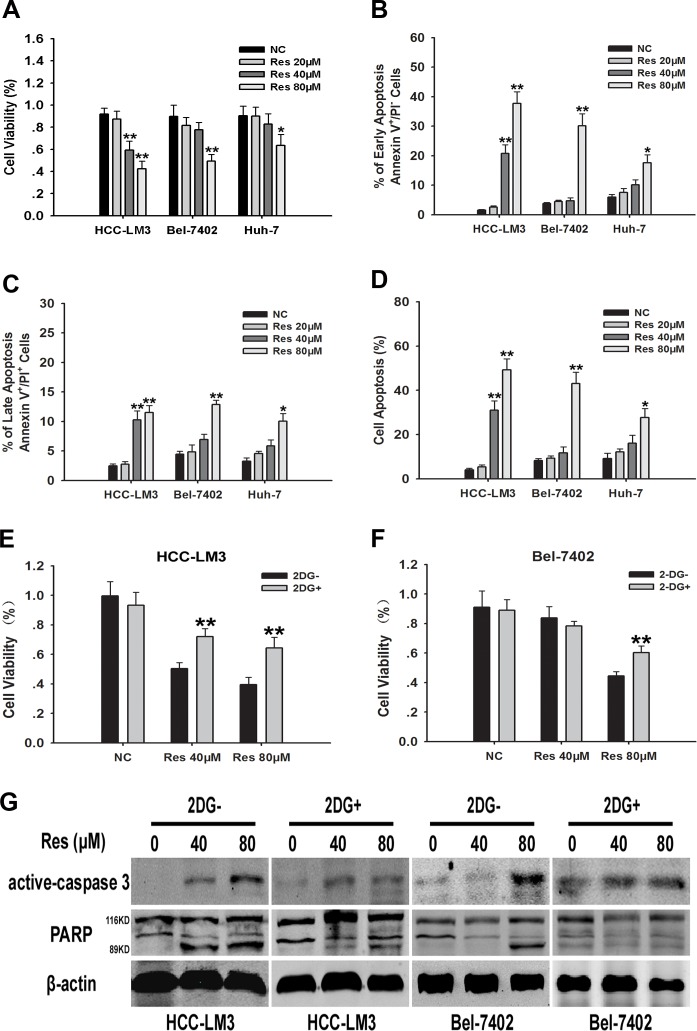
Resveratrol inhibits cell proliferation partly through inhibiton of glycolysis in aerobic glycolytic HCC cells (**A**) HCC cells were cultured with or without resveratrol (20, 40, and 80 μM) for 24 h and cell viability was examined using the CCK-8 method. (**B**) HCC cell lines (5×10^4^) harvested after 24 h of culture with or without resveratrol (20, 40, and 80 μM) were used to examine cell apoptosis by Annexin-V/PI staining. The proportion of cells undergoing the early stage of apoptosis (Annexin-V^+^PI^−^) was indicated. (**C**) The percentage of cells undergoing the late stage of apoptosis (Annexin-V^+^PI^+^) was shown. (**D**) The percentage of cells undergoing both the early and late stages of apoptosis (Annexin-V^+^PI^−/+^) was displayed. (**E**-**G**) 2-DG^−^ or 2-DG^+^ (2 mM) HCC cells were cultured with or without resveratrol (40 and 80 μM) for 24 h. Cell viability and western blot analysis of active caspase-3, β-actin, total-PARP (116KDa) and cleaved-PARP (89KDa) were detected. All experiments were performed in triplicate with similar results (mean ± s.e.m., *P < 0.05; **P < 0.01).

Resveratrol-suppressed glycolysis in tumor cells leads to the inhibition of proliferation in multiple cancers [27, 28]. Therefore, we examined the effect of resveratrol on the proliferation of HCC cell lines. After resveratrol treatment for 24 h, cell proliferation rate was significantly inhibited in a dose dependent manner in HCC-LM3, Bel-7402, and Huh-7 cells, albeit to varying degrees (Fig. 2A). The IC50 of resveratrol for the inhibition of cell proliferation in HCC-LM3, Bel-7402, and Huh-7 cells was 59.46, 81.57, and 111.37 μM, respectively (Fig. 2A).

In addition to the inhibition of cell proliferation, a major effect of resveratrol treatment is the induction of apoptosis in various types of cancer cells [26, 27, 29]. Therefore, we investigated whether resveratrol treatment induced apoptosis in HCC cells. Annexin-V propidium iodide double staining (Annexin-V/PI staining) indicated that resveratrol induced apoptosis in HCC-LM3, Bel-7402, and Huh-7 cells in a dose dependent manner, albeit to varying degrees (Fig. 2B-2D). Resveratrol at a concentration of 40 μM significantly induced apoptosis in HCC-LM3 cells compared to untreated cells, whereas concentrations above 80 μM were required to achieve a similar effect in Bel-7402 and Huh-7 cells. Consistent with the apoptosis staining results, western blotting revealed a significant upregulation of active caspase-3 and cleaved PARP in HCC-LM3 cells treated with 40 μM of resveratrol, whereas a concentration of 80 μM was required to achieve the same effect in Bel-7402 and Huh-7 cells (Fig. S1). These results indicate a window for the selective resveratrol-induced cell apoptosis in HCC cells. In addition, resveratrol enhanced the apoptosis-induced functions in aerobic glycolytic HCC cells.

We next examined whether resveratrol induced cell death was correlated to the inhibition of glycolysis. We hypothesize that glycolysis inhibitors may attenuate the growth inhibitory effects of resveratrol on aerobic glycolytic HCC cells. HCC-LM3 and Bel-7402 cells were treated with resveratrol in the presence or absence of 2-DG, a synthetic glycolysis inhibitor. Resveratrol effectively suppressed the growth of both 2-DG^−^ and 2-DG^+^ cells (Fig. 2E and 2F), although the inhibitory effect of resveratrol was stronger in 2-DG^−^ than in 2-DG^+^ cells, suggesting that cells with a high glycolysis rate are more sensitive to resveratrol. Moreover, 3-bromopyruvate (3-BP), a pyruvate analog that acts as a specific glycolysis inhibitor through a different mechanism of action than 2-DG [30], also attenuated the cell proliferation inhibitory effects of resveratrol in aerobic glycolytic HCC cells (Fig. S2A and S2B). Alternatively, the immunoblotting experiments further demonstrated the signiificant reduction of resveratrol induced active caspase-3 and cleaved PARP in both HCC-LM3 and Bel-7402 receiving the 2-DG (Fig. 2G) and the 3-BP (Fig. S2C). Taken together, these results indicated that resveratrol inhibited cell growth partly through reducing glycolysis in both HCC-LM3 and Bel-7402 cells.

### HK2 is essential for resveratrol-suppressed HCC glycolysis and proliferation

**Figure 3:**
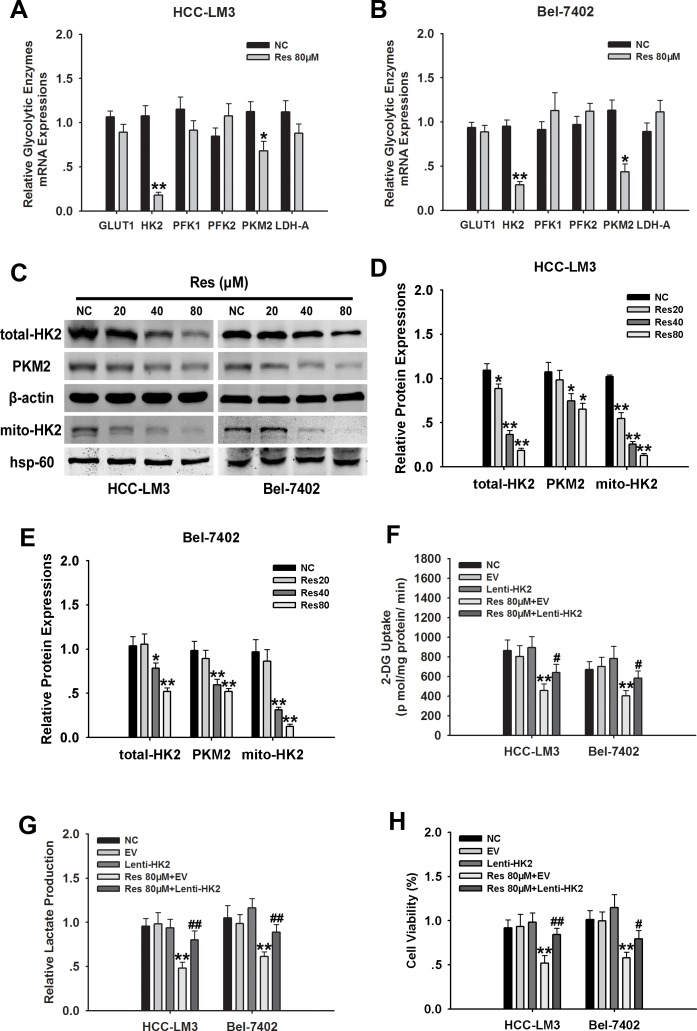
HK2 is essential for resveratrol-inhibited glycolysis and proliferation in aerobic glycolytic HCC cells (**A** and **B**) qRT-PCR analysis of the effect of resveratrol (80 μM) on the expression of genes associated with glycolysis in HCC cells. (**C**–**E**) Western blot analysis of PKM2, β-actin, hsp-60, total and mito-HK2 in HCC-LM3 and Bel-7402 cells treated with or without resveratrol (20, 40, and 80 μM) for 24 h. (**F**–**H**) HCC-LM3 and Bel-7402 were stably transfected with Lenti-HK2 with or without resveratrol 80 μM for 24 h. At the time points indicated, the following measurements were performed: 2-DG uptake (**F**), lactate production (**G**), cell proliferation rate (**H**). Columns represent the mean ± s.e.m. of three parallel experiments (*P < 0.05; **P < 0.01 *vs.* NC group; ^#^*P* < 0.05; ^##^*P* < 0.01 *vs.* resveratrol treatment group).

To examine the mechanism underlying the effect of resveratrol on promoting cell death by inhibiting glycolysis in aerobic glycolytic HCC cells, we measured the effects of resveratrol on the expression of several key glycolytic enzymes, including GLUT1, HK2, PKM2, PFK1/2, and LDH-A in HCC-LM3 and Bel-7402 cells by qRT-PCR. Among them, HK2 mRNA level was decreased the most in both HCC-LM3 and Bel-7402 cells treated with 80 μM resveratrol (Fig. 3A and 3B). Immunoblotting results indicated that HCC-LM3 and Bel-7402 cells treated with resveratrol for 24 h showed a significant decrease in HK2 protein expression in a concentration-dependent manner (Fig. 3C-3E). Importantly, fractionation assays further indicated that in HCC-LM3 and Bel-7402 cells, mitochondrial HK2 was downregulated in response to 24 h of resveratrol treatment, consistent with the pattern of total HK2 (Fig. 3C-3E). Analysis of the potential effect of resveratrol on other glycolytic proteins showed that at a dose of 40 μM, resveratrol downregulated the protein expression of PKM2 in HCC-LM3 and Bel-7402 cells (Fig. 3C-3E), suggesting that resveratrol may inhibit glycolysis at a broad level in aerobic glycolytic HCC cells.

HKs are the first important irreversible enzymes of glycolysis, and the upregulation of mitochondria-bound HK2 in tumor cells promotes cell proliferation and escapes from mitochondria-associated cell death [3, 7, 31]. Therefore, we examined whether resveratrol-downregulated HK2 is essential for resveratrol-inhibited glycolysis in aerobic glycolytic HCC cells. HCC-LM3 and Bel-7402 cells stably overexpressing HK2 (Fig. S3A and S3B) were treated with resveratrol, which significantly attenuated its inhibitory effect on glucose uptake. The inhibitory rate of resveratrol was suppressed from 43.17% and 42.51% in empty vector (EV) groups to 20.44% and 16.83% in HK2 overexpressing groups, respectively. Analysis of lactate levels showed that the inhibitory effect of resveratrol on lactate production was also reduced in HK2 overexpressing groups compared to the EV groups, with inhibitory rates of 50.85% and 37.76% vs. 18.48% and 9.61%, respectively (Fig. 3F and 3G). The cell proliferation rate in HK2 overexpressing-HCC-LM3 and -Bel-7402 cells decreased by 34.48% and 21.68%, respectively, upon 80 μM resveratrol treatment for 24 h (Fig. 3H). These results indicated that HK2 is essential for resveratrol-inhibited glycolysis and proliferation in aerobic glycolytic HCC cells.

### HK2-overexpression in aerobic glycolytic HCC cells attenuates resveratrol-induced mitochondrial apoptosis

**Figure 4:**
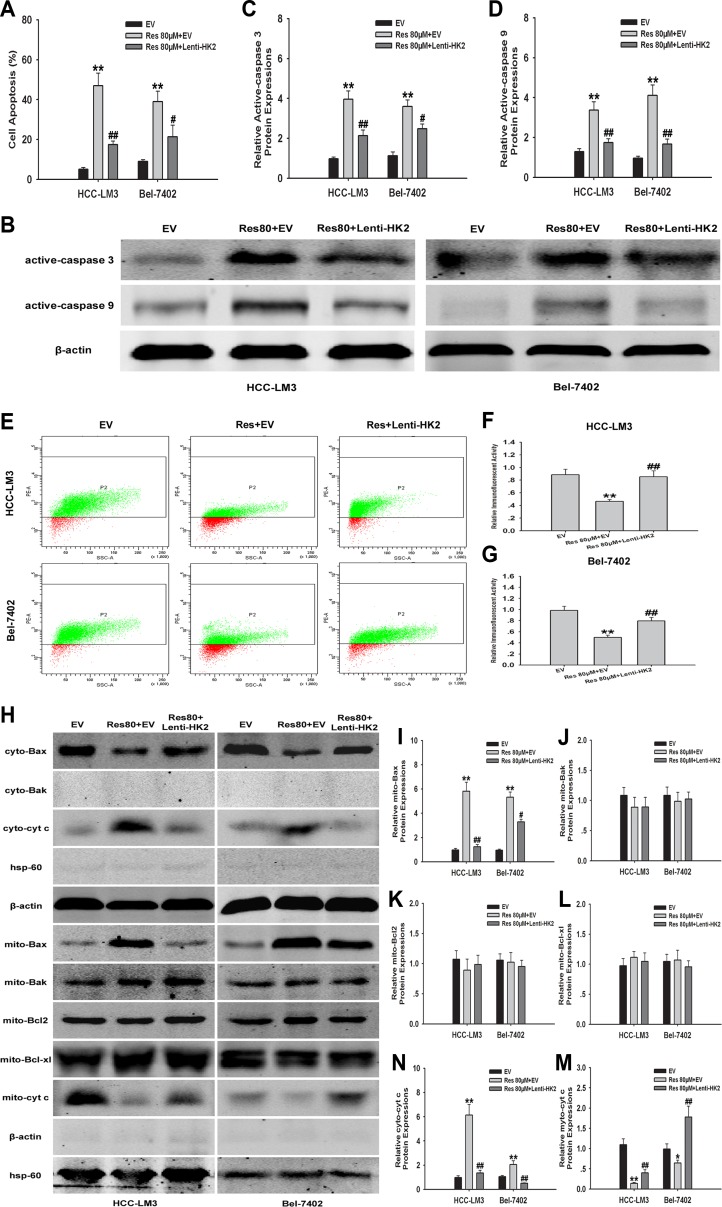
HK2-overexpression in aerobic glycolytic HCC cells reduces resveratrol-induced mitochondrial apoptosis (**A**) EV or Lenti-HK2 cells (5×10^4^) harvested after 24 h of culture with or without resveratrol (80 μM) were used to detect cell apoptosis. Apoptotic cells were tested by Annexin-V/PI staining and the results indicate the proportion of apoptotic Annexin-V^+^PI^+/−^ cells (**P < 0.01 *vs.* EV group; ^#^*P* < 0.05, ^##^*P* < 0.01 *vs.* resveratrol+EV group; means ± s.e.m., n=3). (**B**-**D**) Active caspase-3/9 expression in HCC cells was tested by immunoblotting. The histograms represent the data of three independent experiments (**P < 0.01 *vs.* EV group; ^#^*P* < 0.05, ^##^*P* < 0.01 *vs.* resveratrol+EV group). (**E**–**G**) To measure changes in the Δψm, EV or Lenti-HK2 cells (5×10^4^) treated with or without resveratrol (80 μM) were stained with JC-1 (10 μg/ml) and analyzed by flow cytometry. Green dots represented cells with high Δψm while red dots displayed the low Δψm. Representative histograms show the average of three independent experiments (**P < 0.01 *vs.* EV group; ^##^*P* < 0.01 *vs.* resveratrol+EV group). (**H**-**N**) Cyto- and myto- Bax, Bak, Bcl2, Bcl-xl and chrome c expressions in HCC cells was tested by immunoblotting. Actin and hsp-60 served as loading controls. The histograms represent the results of three independent experiments (*P < 0.05, **P < 0.01 *vs.* EV group; ^#^*P* < 0.05, ^##^*P* < 0.01 *vs.* resveratrol+EV group; means ± s.e.m.).

We next examined the mechanism by which resveratrol induced apoptosis through the downregulation of HK2 in HCC-LM3 and Bel-7402 cells. Resveratrol at a concentration of 80 μM and combined with Lenti-HK2 significantly attenuated resveratrol-induced apoptosis in HCC-LM3 and Bel-7402 cells (17.44% and 21.35% for combination vs. 46.91% and 39.03% for resveratrol alone, respectively) by inhibiting the activation of caspases 3 and 9 (Fig. 4A-4D). In HK2-overexpressing cells treated with resveratrol, Δψm was significantly increased by 39.1% and 29.7% in HCC-LM3 and Bel-7402 cells, respectively, compared to cells treated with resveratrol alone (46.3% and 49.7%; *P* < 0.01, respectively; Fig. 4E-4G). The reduced depolarization effect of Δψm in HK2-overexpressing groups was consistent with a decrease in cytosolic cyt c released from mitochondria to the cytosol (Fig. 4H, 4M and 4N). Additionally, because Bax and/or Bak has been demonstrated to form channels on the outer mitochondrial membrane (OMM) during mitochondrial apoptosis [32, 33], we further tested the subcellular localization of Bax and Bak by immunoblotting using cytosolic and mitochondrial fractions. We found that in resveratrol (80μM) treated group, Bax translocated to mitochondria, while the expression of Bak was not changed in both HCC-LM3 and Bel-7402 cells (Fig. 4H-4J). Moreover, Pastorino JG, *et al* has identified that Bax oligomerization on mitochondria can be attenuated by upregulating mitochondrial binding HK2 [34]. Consistent with their conclusion, by compared to the resveratrol group, we found the significant reduction of Bax oligomerization on mitochondria upon combination treatment with resveratrol and Lenti-HK2 (Fig. 4H and 4I). Alternatively, the expressions of the antiapoptotic protein Bcl2 and Bcl-xl were not altered on mitochondria in both resveratrol and resveratrol combining Lenti-HK2 groups (Fig. 4H, 4K and 4L). These results indicate that resveratrol induced mitochondrial apoptosis in aerobic glycolytic HCC cells through the downregulation of HK2, which probably contributed to the activation and oligomerization of Bax on mitochondria.

### Resveratrol enhances sorafenib induced cell growth inhibition in sorafenib-resistant HCC cells

**Figure 5:**
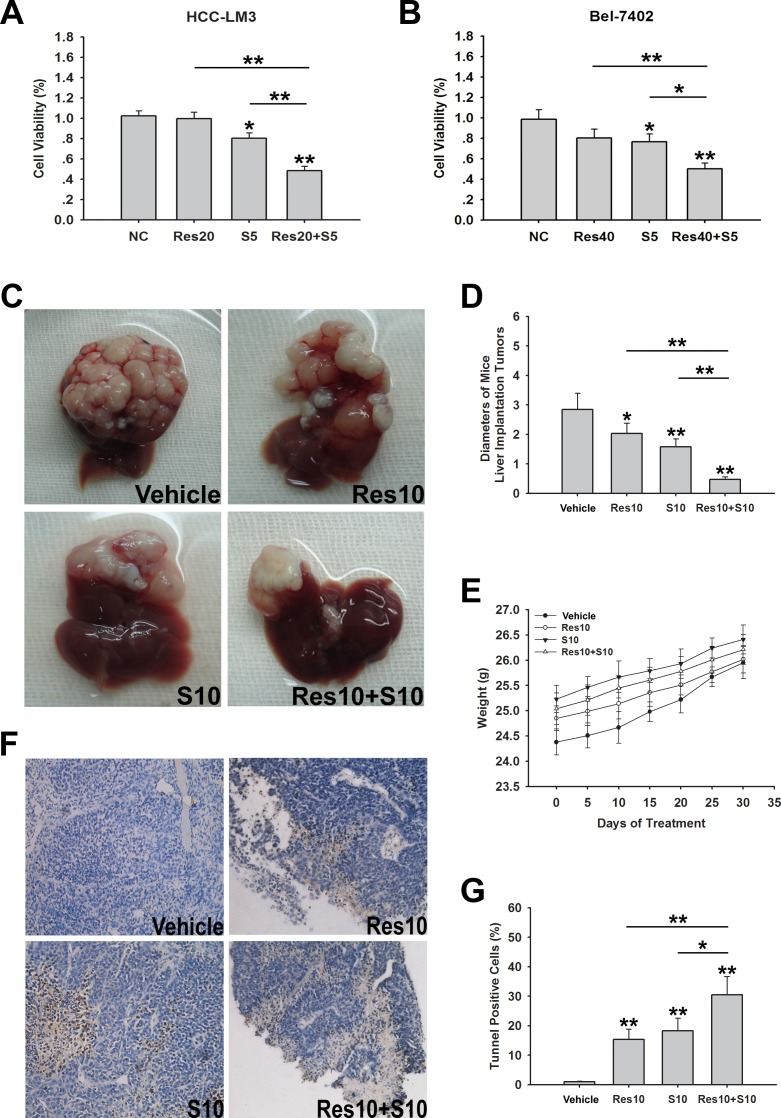
Resveratrol increases sorafenib induced cell growth inhibition in both sorafenib-resistant HCC cells and nude mice bearing liver orthotopic tumor (**A**, **B**) HCC-LM3 and Bel-7402 cells were exposed to resveratrol (20 and 40 μM, respectively), sorafenib (5 μM), and combination treatment for 24 h, and cell proliferation was analyzed using the CCK-8 assay. Columns represent the mean ± s.e.m. of three independent experiments (*P < 0.05; **P < 0.01). (**C**–**G**) In a subcutaneous xenograft mouse model, mice were treated with resveratrol (10 mg/kg BW per day) alone, sorafenib (10 mg/kg BW per day) alone, or combination treatment for 30 days. At the time points indicated, the following measurements were performed: diameters of tumors (**C**, **D**), changes in body weight (**E**), and the percent of TUNEL-positive tumor cells (**F** and **G**). Points or columns represent mean values, whereas bars represent s.d. (*P < 0.05; **P < 0.01).

Our results suggested that resveratrol induces apoptosis and inhibits cell growth via glycolysis inhibition in aerobic glycolytic HCC cells (Fig. 2). Shen et al recently reported that the aerobic glycolysis contributes to sorafenib-resistance in HCC cells, and resistance can be overcome by inhibiting tumor glycolysis [35]. Therefore, we determined whether combination treatment with resveratrol and sorafenib, the only effective anti-HCC drug in clinical practice, had an effect on cell metabolism and cell proliferation in HCC cell lines. The five HCC cell lines tested showed different IC50 values for sorafenib (range: 3.10–20.75 μM; Fig. S4A and S4B). Among them, the two high-glycolytic HCC cell lines HCC-LM3 and Bel-7402, displayed the highest IC50 for sorafenib, which was consistent with the conclusions of a previous report [35]. These cells were regarded as typical sorafenib-resistant HCC cells. HCC-LM3 cell viability was significantly suppressed in response to combination treatment with resveratrol (20 μM) and sorafenib (5 μM) compared with resveratrol or sorafenib alone (Fig. 5A). Consistent with the above results, combined resveratrol and sorafenib (40 and 5 μM, respectively), also markedly decreased the percent of viable Bel-7402 cells compared to the untreated cells (*P* < 0.01; Fig. 5B).

On the basis of the *in vitro* results, we further investigated the anti-HCC activity of resveratrol in nude mice bearing liver orthotopic tumor implants generated by transplantation of glycolytic HCC-LM3 cells. We also determined the combination effects of resveratrol and sorafenib. Mice treated with resveratrol (10 mg/kg/BW/day) or sorafenib (10 mg/kg/BW/day) alone showed a significantly smaller tumor diameter than untreated mice after 30 days of treatment (2.03 ± 0.35, 1.58 ± 0.27 *vs.* 2.84 ± 0.56, *P* = 0.026, *P* = 0.002, respectively; Fig. 5C and 5D). Alternatively, resveratrol combined with sorafenib significantly suppressed tumor size compared with sorafenib alone (0.59 ± 0.09 *vs.* 1.58 ± 0.27, *P* < 0.0001; Fig. 5C and 5D). No significant body weight loss was observed during the 30 days of treatment (Fig. 5E). Compared to the sorafenib group, combination treatment with resveratrol and sorafenib significantly increased the rate of apoptosis (30.46 ± 6.26% *vs.* 18.29 ± 4.27%, *P* < 0.05; Fig. 5F and 5G). Taken together, these results indicate that resveratrol enhances sorafenib induced cell growth inhibition in aerobic glycolytic HCC cell lines and combination treatment with both reagents significantly inhibits tumor growth and induces apoptosis compared with sorafenib alone.

## DISCUSSION

In the present study, we showed that the aerobic glycolysis, which is characterized by enhanced lactate production and glucose uptake, occurred in the majority of the HCC cell lines examined. Resveratrol inhibited cell growth and induced apoptosis by promoting a metabolic shift away from glycolysis in aerobic glycolytic HCC cells. Moreover, we demonstrated that resveratrol downregulates the expression of HK2, a key regulator of tumor glycolysis, which could result in metabolic stress associated apoptosis. The results of the present study showed that resveratrol enhances sorafenib induced cell growth inhibition in aerobic glycolytic HCC cells, and when combined with sorafenib, it increases the *in vivo* effects of sorafenib.

Since the glycolysis can be activated under hypoxic conditions, we hypothesized that HCC cells may not display glycolysis when cultured under normoxic conditions. However, we found that under normoxic conditions, 80% of examined HCC cells displayed the aerobic glycolysis, as indicated by increased lactate production and glucose uptake and decreased oxidative capacity. A low glycolysis in an HCC cell line indicates that either glycolysis is not activated under normoxic conditions or other metabolic pathway, such as oxidative phosphorylation rather than glycolysis, is functional. Our results indicate that glycolysis might influence HCC development, and identify a reagent with few side effects capable of inhibiting the aerobic glycolysis that could be an effective therapy for the treatment of HCC.

In the present study, we showed that resveratrol inhibited glycolysis in aerobic glycolytic HCC cells, as indicated by decreased lactate production and glucose uptake. At the lowest concentration of 20 μM, resveratrol reduced glucose uptake and lactate levels; however, this dose did not significantly suppress the proliferation rate or promote apoptosis, suggesting that glycolysis inhibition by resveratrol in high-glycolytic HCC cells occurs before the inhibition of cell growth and apoptosis induction. In addition, glycolytic inhibitors reduced the sensitivity of aerobic glycolytic HCC cells to the anti-proliferative effects of resveratrol (Fig. 2), suggesting that the resveratrol-inhibited aerobic glycolysis was correlated to cell growth inhibition.

Previous studies have indicated that the reduced glucose uptake induced by resveratrol is the result of decreased glucose transporter expression in lung, breast and leukemic cell lines [36, 37]. However, in the present study, resveratrol had no effect on GLUT1 mRNA expression in aerobic glycolytic HCC cells (Fig. 3). The mechanism underlying the suppression of glucose uptake by resveratrol in HCC cells displaying the aerobic glycolysis remains to be elucidated.

Mitochondria-bound HK2 has been shown to be associated with apoptosis and the escape from mitochondrial cell death in several types of cancer [3, 7, 31]. In the present study, we examined the effect of resveratrol on HK2 correlated glucose metabolism and cell function in aerobic glycolytic HCC cells. Resveratrol treatment had no significant effect on key glycolytic enzymes, such as GLUT1, PFK1/2, and LDH-A (Fig. 3). Although PKM2 was reduced in response to resveratrol, which was consistent with previous research [28], our results also suggested that HK2 is essential for resveratrol correlated metabolic changes and cancer cell growth and apoptosis (Fig. 3 and 4). The present study is the first to indicate that such a mechanism exists in resveratrol-inhibited aerobic glycolysis and cell proliferation in HCC cells. Moreover, HK2-overexpression in aerobic glycolytic HCC cells significantly attenuated resveratrol-induced mitochondrial apoptosis, as indicated by the inhibition of the loss of Δψm and cytochrome c release (Fig. 4).

Sorafenib is the only chemotherapeutic drug that has been shown to be effective in prolonging the survival of HCC patients. However, the low rate of tolerance to sorafenib among HCC patients limits its use [38, 39]. Since several studies have reported that the aerobic glycolysis leads to chemotherapy resistance [40, 41] and glycolysis, which is a potential predictive biomarker of sorafenib resistance [35], is used to overcome sorafenib resistance, we investigated whether inhibition of glycolysis by resveratrol in aerobic glycolytic HCC cells enhances the effect of sorafenib. Two high-glycolytic HCC cells, HCC-LM3 and Bel-7402, showed the highest IC50 for sorafenib among all the HCC cells examined, which was consistent with the conclusions of a previous report [35]. Resveratrol in combination with sorafenib inhibited the proliferation of the HCC cell lines HCC-LM3 and Bel-7402. Furthermore, resveratrol enhanced the ability of sorafenib to reduce the size of tumors in hepatoma-bearing nude mice. These findings are in agreement with previous studies showing that resveratrol can enhance the effect of 5-fluourouracil in colorectal carcinoma [42], clofarabine in malignant mesothelioma [43], ursolic acid in skin tumor cells [44] and tiazofurin in leukemia cells [45].

The present study is the first to investigate the mechanism of resveratrol in glycolysis through the downregulation of HK2 using different HCC cell lines and an HCC-bearing mouse model. HCC is an incurable disease and it is urgent to find new chemotherapeutic options; therefore, it is important to test the potential value of resveratrol in combination with other classic chemotherapeutic drugs such as sorafenib. Our results suggest that resveratrol in combination with sorafenib significantly inhibits the growth and induces apoptosis of HCC-transplanted mice compared to sorafenib alone, providing preclinical evidence of the potential value of this drug for the treatment of HCC. Although the *in vivo* doses of resveratrol used in our study were high, resveratrol is not toxic at high doses in humans [46]. Therefore, in the near future, the prognosis of sorafenib-resistant HCC patients could be improved through the application of the treatment strategy proposed in the present study.

## MATERIALS AND METHODS

### Reagents and cell culture

Resveratrol was purchased from Sigma-Aldrich (St. Louis, MO). Sorafenib tosylate was purchased from Selleck (Selleck Chemicals, Shanghai, China). For cell treatment, the compounds were dissolved in dimethyl sulfoxide (DMSO; Gibco, Carlsbad, CA, USA) and then diluted in media with 10% fetal bovine serum (FBS; Hyclone, Logan, UT, USA) before use.

All cultured cells were purchased from the Chinese Academy of Sciences Committee Type Culture Collection cell bank. HCC-LM3, Huh-7, HepG2, SMMC-7721, Bel-7402, and LO2 were maintained in Dulbecco's modified Eagle's Medium (DMEM) with high glucose (Hyclone) supplemented with 10% FBS. QSG-7701 was cultured in RPMI-1640 with 10% FBS at 37°C in a humidified atmosphere of 5% CO_2_.

### Cell proliferation analysis

HCC cells were cultured with the indicated concentrations of resveratrol for 24 h before the addition of 10 μl CCK-8 solution (Peptide Institute Inc., Osaka, Japan) to each well. The plate was maintained in the incubator for 4 h. The absorbance was measured at 450 nm using a microplate reader. The IC50 values were calculated using CompoSyn software using the absorbance data obtained with a microplate reader Model 680 (BIO-RAD, Hercules, CA).

### 2-DG uptake, lactate production, and O_2_ consumption

After washing the cells twice with the uptake buffer (140 mM NaCl, 2 mM KCl, 1 mM KH_2_PO_4_, 10 mM MgCl_2_, 1 mM CaCl_2_, 5 mM glucose, 5 mM L-alanine, 5 mM indomethacin, and 10 mM HEPES/Tris, pH 7.4), cells were cultured in uptake buffer containing 1 μCi/mL [^3^H]-2-DG at 37°C for 30 min. After 30 min, the cells or tumor tissues were solubilized by 0.1% Sodium Dodecyl Sulfate (SDS) and the radioactivity was counted using a liquid scintillation counter. The radioactivity counts for each sample were then normalized to the protein level and corrected for the zero-time uptake per mg protein. Lactate levels were measured by a fluorometric assay (BioVision, Milpitas, CA, USA) according to the manufacturer's protocol. O_2_ consumption was tested using the 110 Fiber optic oxygen monitor (Instech, Plymouth Meeting, PA). Approximately 5 million cells were collected in 500 μl of media and cultured at 37°C. Results were tested in three independent experiments and displayed as nmol O_2_/million cells/min.

### Reverse transcriptase-polymerase chain reaction (RT-PCR) and quantitative real-time PCR (qRT-PCR)

The Trizol reagent was used to extract total RNA according to the manufacturer's protocol. Total RNA (2,000 ng) in each sample was used to generate cDNA using SuperScript II reverse transcriptase with Oligo (dT) (Invitrogen, Carlsbad, CA).

The real-time PCR experiment was performed following the protocol of the real-time PCR kit (Takara, Dalian, China) [18-19].

### Western blot analysis

The cytosolic and mitochondrial fractions were separated and purified from HCC cells using a Mitochondrial Isolation Kit (Pierce, Rockford, IL, USA) according to the manufacturer's protocol [20].

Total cellular proteins were extracted using radio-immunoprecipitation assay (RIPA) buffer (Sigma-Aldrich). For reducing gel electrophoresis, equal amounts of samples were added to each lane. The samples were then resolved by SDS-PAGE and transferred to polyvinyl difluoride membranes. The membranes were sequentially blocked with 5% defatted milk, and incubated overnight with the following primary antibodies: β-actin (A2228) (1:2,000; Sigma-Aldrich); HK2 (#2867) (1:1,000; Cell Signaling Technology, Beverly, Mass); PARP (13371-1-AP), Bax (23931-1-AP), Bak (14673-1-AP), Bcl-2 (12789-1-AP) and Bcl-xl (10783-1-AP) (1:500; Proteintech Group, Chicago, IL); active-caspase 3 (ab32042), active-caspase 9 (ab2324), cytochrome c (cyt c) (ab13575), and hsp-60 (ab46798) (1:1,000; Abcam, San Francisco, CA). The OXPHOS cocktail (MS604) (1:250; Mitoscience, Cambridge, Massachusetts, USA) was used against the following proteins: NDUFB8 of complex I (20 kD), SDHB of complex II (30 kD), UQCRC2 of complex III (48 kD), MTCO1 of complex IV (40 kD), ATPSA of complex V (55 kD). The LI-COR ODYSSEY scanner (LICOR) was used to detect the antigens on the blots.

### Annexin-V/PI staining

HCC cells were cultured in six-well plates and treated as indicated. After 24 h, the cells were centrifuged, washed twice with PBS, and mixed in 100 μl of 1× binding buffer (10mM HEPES/NaOH, PH7.4, 140mM NaCl, 2.5mM CaCl_2_). After culturing for 15 min at room temperature in Annexin-V/PI (BD Biosciences, San Jose, CA) double staining liquid, the cells were examined by flow cytometry (Cytomics FC500; Beckman Coulter, Fullerton, CA). The percentage of apoptotic cells was calculated using ModFitLT software.

### JC-1 staining to measure mitochondrial membrane potential

Cells were plated on coverslips with different treatments overnight. After staining with 10 μg/ml JC-1 (JC-1 Mitochondrial Membrane Potential Detection Kit; Invitrogen) for 15 min in the incubator (37°C, 5% CO_2_), cells were rinsed with HBSS twice to remove the non-specific background staining. Cells were analyzed using a flow cytometer (Beckman Coulter), and cells emitting a bright red fluorescence represented the aggregate mitochondria.

### Plasmid construction, lentivirus packaging and infection

To construct the lentivirus vector pCDH-HK2 expressing HK2, a full-length cDNA encoding the HK2 sequence was amplified from 293T cDNA and then cloned into the EcoR I/BamH I sites in the pCDH-CMV-MCS-EF1-GFP vector (System Biosciences, Mountain View, CA). Control lentiviral vector was used as a control. All plasmid sequences were confirmed by DNA sequencing. Lentivirus was generated by co-transfecting pCDH-CMV-MCS-EF1-GFP empty vector or pCDH-HK2 in 293T cells with Lipofectamine™2000 (Invitrogen). Target cells were infected overnight with empty vector or pCDH-HK2 in the presence of 8 μg/ml polybrene (Sigma-Aldrich). The following day, the cells were given fresh medium and allowed to grow for another 48 h. The transduction efficiency was measured by western blotting. The sorted cells were then characterized and used in further assays.

### Animal experiments and ethics statement

Four-week-old male athymic BALB/C nu/nu mice with free access to water and food were housed in a standard animal laboratory with a 12-h light-dark cycle and constant environmental conditions. All experiments were performed in accordance with ethical standards and in compliance with the Declaration of Helsinki, and according to national and international guidelines. The study was approved by the Animal Care and Use Committee of Shanghai Tongji University. Serum-free culture medium (300 μl) containing HCC-LM3 cells (5×10^6^) was subcutaneously injected into the upper flank region of three mice. After one week, 1.0 mm^3^ subcutaneous tumor tissue was placed in the livers of other mice as described previously [21-23]. Twenty mice were randomly divided into four groups, namely vehicle, resveratrol, sorafenib, resveratrol+sorafenib. Resveratrol was suspended in DMSO and diluted with physiological saline containing 1%DMSO. Mice in the resveratrol group were treated by intraperitoneal injection once daily for 30 days with resveratrol (10 mg/kg) dissolved in 1%DMSO/saline solution. Thus 1%DMSO/saline served as an intraperitoneal vehicle. Sorafenib was dissolved in an oral vehicle containing Cremophor EL (Sigma-Aldrich), 95% ethanol and water in a ratio of 1:1:6 as described previously [24], and orally administrated at a dose of 10 mg/kg by gavage daily for 30 days. Mice in the vehicle group received oral and intraperitoneal vehicles; mice in the resveratrol group, oral vehicle and intraperitoneal resveratrol; mice in sorafenib group, intraperitoneal vehicle and oral sorafenib; and mice in resveratrol+sorafenib group, intraperitoneal resveratrol and oral sorafenib. During this time, mice were weighed every 5 days. At the end of the experiment, the mice were killed by cervical dislocation. Livers were resected and imaged using a high-definition digital camera.

### TUNEL assay

Apoptosis of tumor tissues was assessed using the TUNEL assay. Paraffin-embedded sections (5 μm) were cut and mounted on glass slides. The sections were deparaffinized and then digested with 20 μg/mL proteinase K (Sigma-Aldrich) for 15 min at room temperature. The slides were washed 4 times in distilled water for 2 min, incubated with 2% hydrogen peroxide in PBS for 5 min at room temperature, washed with PBS twice and immersed in TdT-containing buffer for 15 min to prepare digoxigenin-binding sites. An anti-digoxigenin antibody fragment carried a conjugated reporter enzyme (peroxidase) to the reaction sites, and then localized peroxidase generated an intense signal from the chromogenic substrate diaminobenzidine. The counterstain was methyl green.

### Statistical analysis

Statistical analysis was performed with SPSS 17.0 software and performed using a two-tailed unpaired Student's t-test. Quantitative data are representative of at least three independent experiments. Values of *,^#^*P*<0.05 and **,^##^*P*<0.01 were considered statistically significant.

## SUPPLEMENTARY MATERIAL AND FIGURES


