# Supplementary material for: By reducing hexokinase 2, resveratrol induces apoptosis in HCC cells addicted to aerobic glycolysis and inhibits tumor growth in mice
Source: Oncotarget. 2015 Apr 12;6(15):13703–17. doi: 10.18632/oncotarget.3800 (PMC4537043; doi:10.18632/oncotarget.3800)
Supplement: Supplementary file 1 [file oncotarget-06-13703-s001.pdf]

By reducing hexokinase 2, resveratrol induces apoptosis in HCC cells addicted to aerobic glycolysis and inhibits tumor growth in mice

Supplementary Material

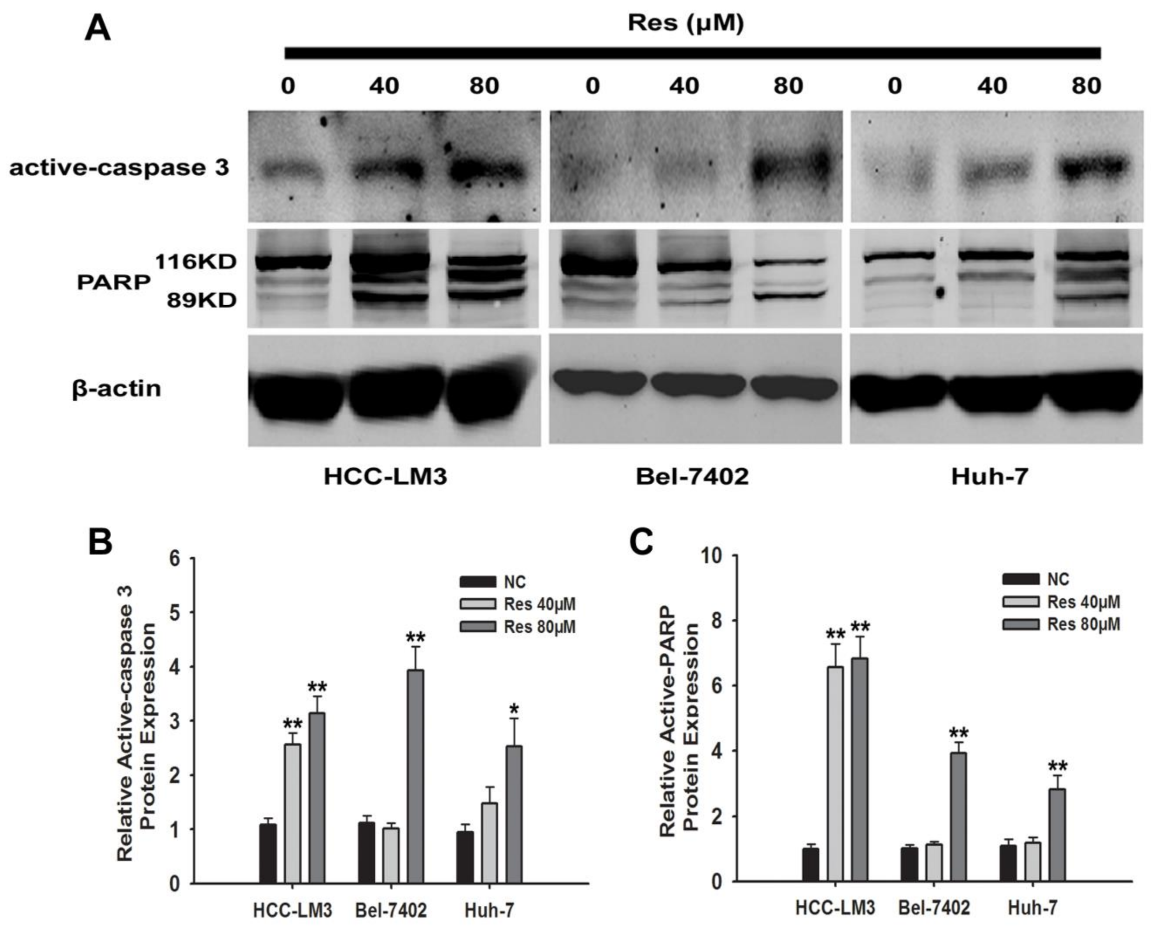

Figure S1: Active caspase-3, total-PARP (116KDa) and cleaced-PARP (89KDa) protein expressions in HCC cells was detected by immunoblotting. The histograms represent the results of three independent experiments (mean ± s.e.m., **P* < 0.05; ***P* < 0.01 *vs.* NC group).

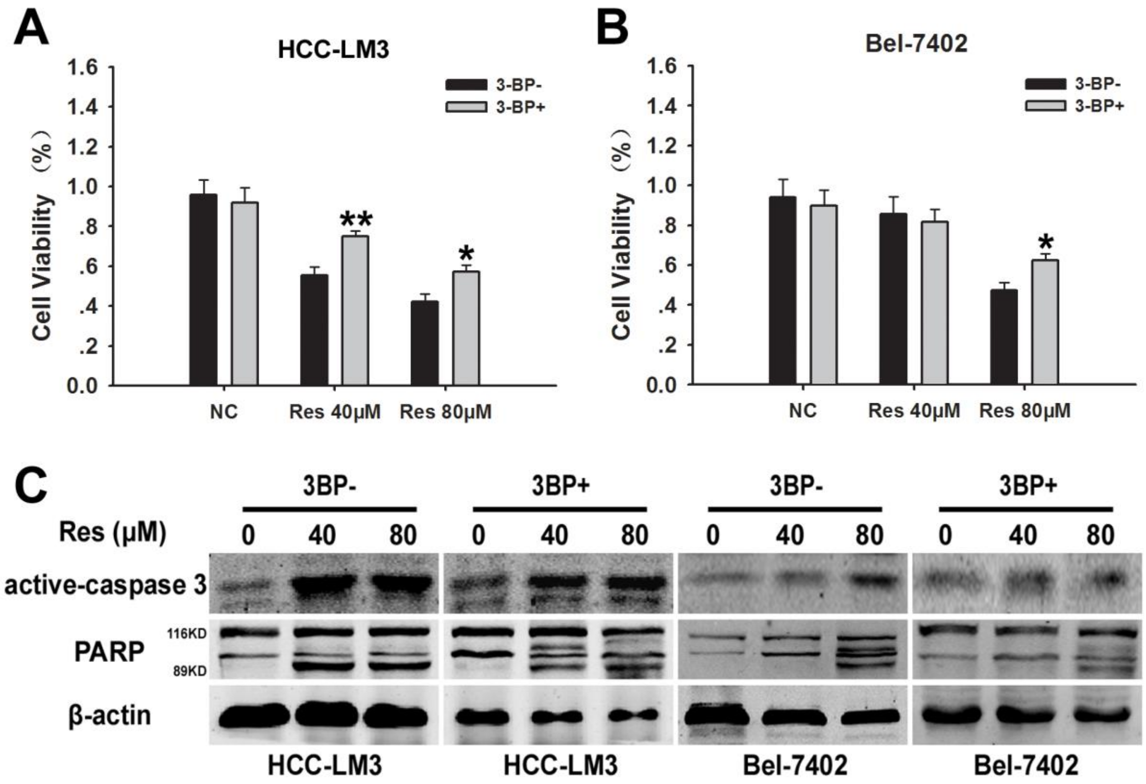

Figure S2: 3-BP$^-$ or 3-BP$^+$ (100 μM) HCC cells were cultured with or without resveratrol (40 and 80 μM) for 24 h. (A-C) Cell viability and western blot analysis of active caspase-3, β-actin, total-PARP (116KDa) and cleaved-PARP (89KDa) were examined. The results represent the mean ± s.e.m. of three independent experiments (*P < 0.05; **P < 0.01).

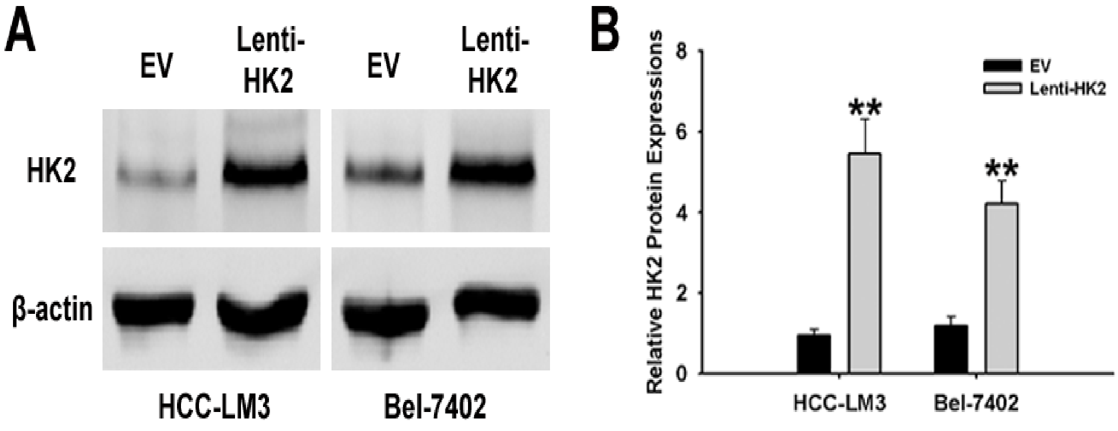

Figure S3: Transduction efficiency of HK2 overexpression in HCC cells. (A, B) HK2 and β-actin were analyzed by immunoblotting after HCC cells were transduced by lentivector encoding HK2. Images are representative of three independent experiments. Columns represent the mean ± s.e.m. (***P* < 0.01).

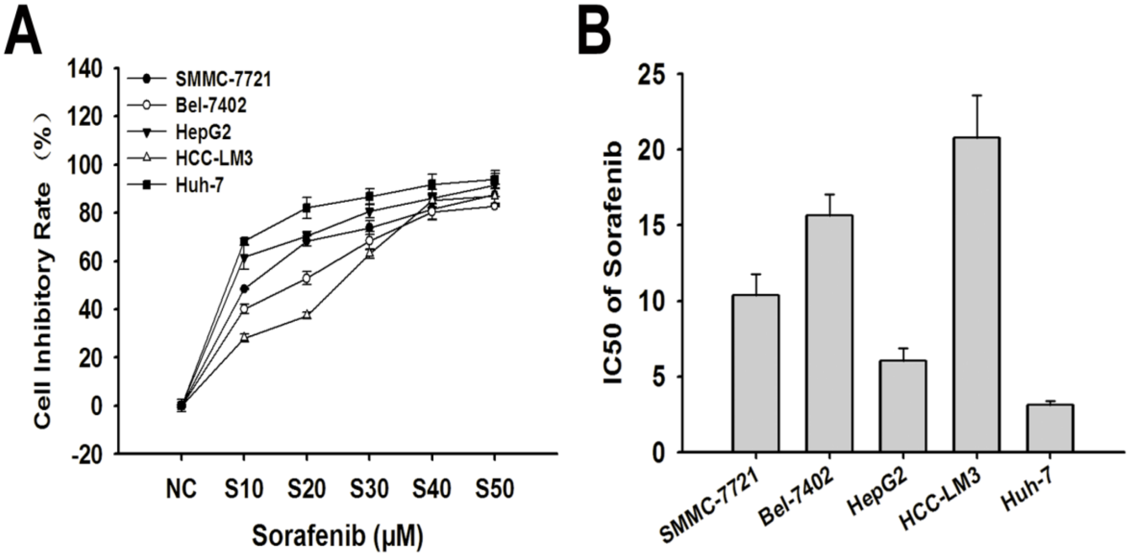

Figure S4: Calculation of IC50 values for sorafenib in a panel of HCC cells. (A and B) After 24 h of culture with or without sorafenib (10-50 μM), HCC cells (5×10^4) were harvested and analyzed for cell growth inhibition using the CCK-8 assay. The IC50 values for sorafenib were calculated based on the changes in absorbance, as determined using a microplate reader. Columns represent the mean ± s.e.m of three independent experiments.
